# Celebrating the achievements of a Nordic journal on social medicine and public health

**DOI:** 10.1177/14034948221094443

**Published:** 2022-05-21

**Authors:** Charli Eriksson

The start of Nordic collaboration in publishing an international journal on social medicine and public health was marked by the emergence of the Nordic Social Medicine Association in 1966 and by the joint efforts of institutions of social medicine in the five Nordic countries of Denmark, Finland, Iceland, Norway and Sweden. At the first Nordic Congress on Social Medicine, held in Gothenburg, Sweden, in 1967, it was agreed that that the country associations should jointly publish an international scientific journal [1]. The congress embraced proceedings on a broad range of issues, including epidemiology in relation to internal medicine, psychiatry, paediatrics, traffic medicine and other branches of medicine, as well as applied social medicine, and also a symposium on epidemiological aspects of cerebrovascular diseases.

The first issue of *Acta Socio-Medica Scandinavia* appeared two years later. The programme and vision of the journal were presented in the Editor’s introduction [2]:

Social medicine is not a new science. It has always been the custom of medical practitioners to observe the sick individual in the framework of his social surroundings. In relieving sickness and need, the practitioner often has to catch up with shortcomings brought about by the structure of society. . . . Social medicine concerns those factors, among individuals as members of different social groups, that . . . in conjunction with factors in the structure of society affect the condition of general health. Any change in the factors that improve the state of health of individuals and groups are included here. . . . In conclusion, medical research is designed to improve the living conditions of mankind, and thereby society itself. In the evolution of the welfare state, it has important duties and tasks.

The Editor noted that, ‘modern social medicine involves close contact with various other disciplines. . . . Both research work and everyday practical activities have as a rule to be carried out by experts working together in teams’.

After four annual volumes and six supplementary monographs, it was decided that the journal should be changed in design, publishing house and name. The name of the journal was changed to the *Scandinavian Journal of Social Medicine*, but the programme of the journal remained unchanged [2]. The first editorial stated that, ‘with the structural rationalisation of working life, environmental abuse, urbanisation, and other prominent features of social progress, the need for measures to prevent disease and behavioural deviations is more urgent than before’.

Twenty years after the start of the Nordic journal, an editorial noted that most authors emphasised the social causes and consequences of disease and other aspects of medicine [3]. There was a call for help from researchers to discuss central themes in public health, namely, ‘the influence of social factors on health, and the influence of disease in everyday life’. The journal could be an important voice in the formulation of strategies for evidence-based disease prevention and health promotion.

Over time, the concept of ‘social medicine’ has changed meaning [4]. The broad concept was thought as ‘a rootstock nurturing fresh plants in the public health garden’. Plants like epidemiology, health services research, health economics, health and social policy studies, preventive medicine, health promotion, community health, health psychology and medical sociology all belong to the same family. It had increasingly been recognised that major health problems cannot be understood and solved within a narrow biomedical paradigm. The Editors also stated that the term ‘medicine’ should not discourage non-medical professional groups from using the journal.

After 30 volumes, the *Scandinavian Journal of Social Medicine* became the *Scandinavian Journal of Public Health* [5]. This was the outcome of a year-long discussion in the Nordic Association of Social Medicine, which oversaw the development of the journal over the years into a multidisciplinary forum for public health sciences and practice. The journal had a change in name, but not in vision. In Sweden, a section for social medicine had been created in 1923 within the Swedish Society of Medicine. The first Chair in Social Medicine was established at the University of Oslo in 1951. The new discipline was inspired by the development of British social medicine in the 1940s and nation-level studies of the relationship between health, disease and socioeconomic factors. Scandinavian academic social medicine also developed a distinct micro-level or clinical approach to the study of the socioeconomic determinants and consequences of disease in individuals and families. However, group-based and clinical social medicine was ‘overshadowed by a strong growth in epidemiology and health services research and the parallel movement of social science research into the field of health’ [5]. The Nordic university departments in social medicine expanded during the 1980s and 1990s into larger multidisciplinary institutions for public health sciences.

Public health has roots in the sanitary movement, with a primary orientation towards environmental factors. The epidemiological transition led to a focus on behaviour-related chronic diseases, but public health vision and practice were undermined by the individualisation of population-wide health problems. There was a growing awareness that population health cannot be achieved with curative medicine alone. The new public health combined ‘the best of the hygienic and sanitary tradition with a participatory approach to ecological health development’ [6]. The biomedical model was regarded as clearly insufficient in itself. Public health is more than a branch of medicine and the art of public health action depends on contributions from many different professions and fields of activity. The basic vision of the journal lives and drives on the earlier visions of the founding fathers of social medicine and public health from the 19th century. The journal got a new name and format, and new editors.

Public health has shown a dramatic improvement in many indicators of population health [7] and people in the Nordic countries are among the winners. General improvements are seen in health, functional ability and health-related quality of life. Specific public programmes, such as vaccination and prevention programmes related to reproduction and child health, have also contributed to good health. The new Chief Editor, Professor Stig Wall [7], noted several challenges: the health transition (to the chronic disease of ‘welfare’ and ageing), equity in health (on the public health agenda in most countries and agencies) and the shift from individual risk factors to structural, methodological development (involving conceptual and methodological contributions from epidemiologists, health economists and sociologists). All this implies ‘placing greater importance on the health effects of socioeconomic and environmental change, developing theoretical and methodological measures of outcomes in preventive programmes, analysing the social consequences of community-oriented health work, and assessing the preventive potential of health care involvement in public health interventions’. The journal can influence health research disequilibrium – that too little research effort is addressed at the bulk of current health problems. The Editors commented on the process in an editorial, where the goals of the journal were described in terms of broadening the scope of the journal to make it a multidisciplinary forum for public health research and policy [8]. The publishing of supplements on important policy issues has made a significant contribution over the years. An analysis covering the years 2000–2004 showed that papers on child and adolescent health were the most prevalent, followed by those on socioeconomics and inequality [9].

In 2008, the journal entered a new era [10] and was published eight times a year. The new publishing house is SAGE, following a bidding process, and accepted articles now appear on SAGE’s OnlineFirst platform. Director Finn Kamper-Jørgensen became the Chief Editor and a new section on policy statement reviews from the Nordic Ministers for Health was introduced. The intention to be an international forum for Nordic as well as international public health research and policy was emphasised by the new Chief Editor. The vision was the same, but an additional emphasis was to ‘contribute to an open and better dialogue between researchers, practitioners, administrators and policymakers in public health’. A good international journal of public health depends on high-quality contributions from researchers and other persons interested in public health development. However, it also depends on the willingness of reviewers to conduct evaluations of scientific manuscripts, something that can lead to long review processes [11].

In 2012, the Chairman of the Executive Board presented a call for a new Chief Editor [12]. It was stated that the journal wished to celebrate its 40th anniversary from its origin as the *Scandinavian Journal of Social Medicine*. The journal was then an important forum for health-oriented welfare research. From an international perspective, the Nordic countries have unique opportunities for register-based and linked register-based research. This potential for Nordic research can be further extended and deepened [13]. Professor Ingvar Karlberg at the Nordic School of Public Health was elected as Chief Editor [14] and the content of the journal was totally determined by input from researchers. The new Editor was interested in including more scientific assessments of policy in public health. He was eager to speed up the peer review process [15] and improve the quality of the journal [16]. The Nordic School of Public Health was a unique Nordic institution started in 1953 by the Nordic countries because of the lack of postgraduates concerned with public health and health services. The degree of Master of Public Health was awarded from 1978 and the first Doctor of Public Health graduated in 1987. However, the Nordic School of Public Health had to close [17], creating the need for a new home base for the journal.

From 2016, the journal has been printed and distributed on paper, but it is also available in a purely digital format [18]. The journal publishes research on the relationship of society and health to its determinants and many studies are theoretically inspired by, and emanate from, the Nordic Welfare Model. Its finances are stable. The new Chief Editor, Professor Terje Andreas Eikemo, took over responsibility for editorial work in 2017, with a sharper focus on the most pressing public health challenges in the Nordic countries and beyond [19]. Greater attention is paid to social injustice as a driver of health inequalities, which is ‘above all a matter of the wider social, economic, and cultural circumstances in which we are born, grow, live, work, and age’. Health inequalities are socially produced, they are potentially avoidable and they are widely regarded as unacceptable.

The new Chief Editor developed an editorial board consisting of five Nordic researchers covering five key research areas [19]: migration/ethnicity/refugee health (Denmark), occupational health (Finland), mental health (Iceland); ageing and health (Norway) and global health/child health (Sweden). The journal encourages contributors ‘not only to take an interdisciplinary approach involving, for example, sociology, psychology, technology, social medicine, but also an intersectoral approach, including of course academia, but also people working in government, NGOs, think-tanks, the UN, and health care’.

Development in the universities can be regarded as an increasingly specialised activity where research approaches are influenced by how attractive the questions are for publishing and referencing. In the social sciences, this has been described as a transition from science as a vocation to science as a game [20]. The concern of some academics is to get published rather than having something socially meaningful to say. Higher education has been transformed from a temple of wisdom to a factory producing academic credits for the many. The public health sciences and this journal have important messages for policy-makers, practitioners, academic professionals and the public. Since it started in 1969, the journal has been vital in this regard.

Modern science has a tendency towards specialisation and fragmentation, but interdisciplinary science is a multifaceted phenomenon, which can counteract these trends. Disciplinary boundaries are constituted by differences in theory and method and have, in part, an organisational, social and cultural dimension. Public health and social medicine have always been an antidote to fragmentation and enhance knowledge integration. Integration is what is sought after in interdisciplinary science. It is about what should be integrated (theories, methods, concepts), how the integration should be achieved, and by whom [21].

Three types of analysis are typically distinguished in interdisciplinary research. The first is the multidisciplinary type, where research in a problem area is performed using the methods of several individual disciplines in parallel: side-by-side, but largely independent of each other. The second is the interdisciplinary approach, where researchers from two or more disciplines collaborate in areas that overlap in a disciplinary or methodological way. This type of research requires a shared problem formulation and, to some extent, a shared approach to methodological research. The third is the transdisciplinary path, which transcends disciplinary boundaries. It involves the development of a common language and new or unique methodologies. Trans-disciplinarity has four characteristics [22]: (1) it develops a framework for guiding problem-solving efforts; (2) it develops its own distinct theoretical structures, methods and practical approaches; (3) it communicates beyond institutional pathways directly to those who participate in the research; and (4) it is dynamic with less predictable processes than disciplinary research. Also, collaboration takes place both between researchers from different disciplinary backgrounds and between researchers and practitioners. The emphasis in social medicine and public health sciences has always been on joining the forces of researchers from different disciplinary backgrounds.

The focus of the journal on social justice as a driver of health inequality requires transdisciplinary development to approach the vision of contributing to health for all in the population. Public health needs to be evidence-based, which means that learning and knowledge production for public health must be comprehensive and include knowledge from different domains [23]: the distribution of health, determinants or causal web, consequences, intervention methods and policy options. The fundamental challenge for the future is to do the right things in the right way. This requires clear priorities. What are the important issues for analysis, advocacy and action? Public health activities need to be grounded in political decisions and ethical considerations. The journal has been a key actor in the endeavour to bridge the gap between research and action.

It is time to celebrate the achievements of the journal after more than 50 years since the start of the Nordic collaboration that enabled its publication – acknowledging the past, reflecting on present challenges and considering the potential of public health science and practice to produce a better future life for all.


Charli Eriksson, Professor Emeritus in Public Health Sciences Co-Editor of the *Scandinavian Journal of Public Health*, 2008–2016

